# Dengue, Yellow Fever, Zika and Chikungunya epidemic arboviruses in Brazil: ultrastructural aspects

**DOI:** 10.1590/0074-02760200278

**Published:** 2021-02-03

**Authors:** Debora Ferreira Barreto-Vieira, Dinair Couto-Lima, Fernanda Cunha Jácome, Gabriela Cardoso Caldas, Ortrud Monika Barth

**Affiliations:** 1 Fundação Oswaldo Cruz-Fiocruz, Instituto Oswaldo Cruz, Laboratório de Morfologia e Morfogênese Viral, Rio de Janeiro, RJ, Brasil; 2 Fundação Oswaldo Cruz-Fiocruz, Instituto Oswaldo Cruz, Laboratório de Mosquitos Transmissores de Hematozoários, Rio de Janeiro, RJ, Brasil

**Keywords:** arboviruses, transmission electron microscopy, viroplasm, flaviviruses

## Abstract

**BACKGROUND:**

The impact of arbovirus cocirculation in Brazil is unknown. Dengue virus (DENV) reinfection may result in more intense viraemia or immunopathology, leading to more severe disease. The Zika virus (ZIKV) epidemic in the Americas provided pathogenicity evidence that had not been previously observed in flavivirus infections. In contrast to other flaviviruses, electron microscopy studies have shown that ZIKV may replicate in viroplasm-like structures. Flaviviruses produce an ensemble of structurally different virions, collectively contributing to tissue tropism and virus dissemination.

**OBJECTIVES AND METHODS:**

In this work, the *Aedes albopictus* mosquito cell lineage (C6/36 cells) and kidney epithelial cells from African green monkeys (Vero cells) were infected with samples of the main circulating arboviruses in Brazil [DENV-1, DENV-2, DENV-3, DENV-4, ZIKV, Yellow Fever virus (YFV) and Chikungunya virus (CHIKV)], and ultrastructural studies by transmission electron microscopy were performed.

**FINDINGS:**

We observed that ZIKV, the DENV serotypes, YFV and CHIKV particles are spherical. ZIKV, DENV-1, -2, -3 and -4 presented diameters of 40-50 nm, and CHIKV presented approximate diameters of 50-60 nm. Viroplasm-like structures was observed in ZIKV replication cycle.

**MAIN CONCLUSIONS:**

The morphogenesis of these arboviruses is similar to what has been presented in previous studies. However, we understand that further studies are needed to investigate the relationship between viroplasm-like structures and ZIKV replication dynamics.

Currently, the Brazilian public health system is going through a challenging scenario due to arboviruses. As dengue virus (DENV) has a hyperendemic profile, it has resulted in the cocirculation of the four DENV serotypes,^1^ the emergence of Zika virus (ZIKV)^2^ and Chikungunya virus (CHIKV),^3^ and the risk of reurbanisation of Yellow Fever virus (YFV).^4^ Vector control remains the sole effective method of prevention since there are no effective vaccines or antivirals for all arboviruses in question.^5^ ^,^ ^6^

The perplexity of the dissemination of ZIKV and CHIKV and their impact in Brazil was enough to declare a public health emergency by the Ministry of Health and the World Health Organization almost two years after the introduction of these viruses in Brazil. The intense mobilisation of resources and cooperation between states and municipalities to confront the viral circulation has become a grand undertaking.^7^

DENV (consisting of four antigenically different serotypes: DENV-1, DENV-2, DENV-3 and DENV-4), YFV and ZIKV belong to the genus *Flavivirus*, *Flaviviridae* family*.* The virus particles are round with an approximate diameter of 60 nm and have a lipid bilayered envelope that comprises the E protein (envelope protein) and the prM protein (precursor membrane protein). The prM protein is cleaved into the M protein during exocytosis, thus ensuring the infectivity of the viral particle. The icosahedral nucleocapsid consists of a positive-sense single-stranded ribonucleic acid chain (ssRNA) of ~10.7 kb and the C protein (capsid protein) has a diameter of approximately 30 nm. The mechanism of ZIKV replication has not been well studied in detail thus far. A previous study showed that part of the replicative cycle might occur within a viroplasm-like cell compartment, a structure never observed before in other flavivirus replication cycles.^8^

CHIKV belongs to the *Alphavirus* genus within the *Togaviridae* family. Alphaviruses are enveloped spherical particles with a diameter of 65-70 nm,^9^ ^,^ ^10^ and their genome consists of a single-stranded positively sensed 11.8 kB RNA molecule packaged by the C protein to form the nucleocapsid. This nucleocapsid is surrounded by a host cell-derived lipid bilayer with two inserted transmembrane glycoproteins, E1 and E2.^11^ The composition of the host cell-derived lipid bilayer strongly resembles the plasma membrane of the infected host cell.^12^

In this study, the morphology and morphogenesis of these four arboviruses currently circulating in Brazil were analysed using transmission electron microscopy.

## MATERIALS AND METHODS

Virus samples

*Dengue viruses* - The DENV-1, -2, -3 and -4 samples used in this study were isolated in C6/36 cell culture by the Laboratório de Flavivirus, Instituto Oswaldo Cruz (IOC), Fundação Oswaldo Cruz (Fiocruz) from sera obtained during epidemics in the state of Rio de Janeiro, Brazil in 2008, 2000, 2008 and 2013, respectively. The samples were previously serotyped by indirect immunofluorescence using DENV-specific monoclonal antibodies against DENV-1: 15F3, DENV-2: 3H5, DENV-3: 8A1 and DENV-4: Ascitic fluid Anti Dengue 4^13^ and by real-time quantitative polymerase chain reaction (RT-qPCR) using specific primers.^14^ The viral titres were DENV-1: 10^6.075^ PFU/mL, DENV-2: 10^6.51^ PFU/mL, DENV-3: 10^7.08^ PFU/mL and DENV-4: 10^8.85^ PFU/mL. The viral stock was stored at -80ºC.

*Yellow Fever virus* - The YFV sample used was the 74018 isolate belonging to South American genotype I, isolated from Brazil, corresponding to the distinct lineage 1D 74018/FIOCRUZ/MG/01 (YFV-74018-1D) from a human fatal case in 2001. The isolate was passaged four times in C6/36 cell culture and grown in T25 flasks in Leibovitz medium (L-15) (Invitrogen) supplemented with 10% foetal bovine serum (FBS) (Gibco) and maintained at 28ºC to produce large quantities of infected supernatant. The viral stocks were stored at -80ºC until use. The viral titre was 6.5 x 10^6^ PFU/mL. The sample was provided by the Laboratório de Mosquitos Transmissores de Hematozoários/LATHEMA, IOC/Fiocruz.

*Zika virus* - The ZIKV sample used was isolated in C6/36 cells from Brazilian patient blood in 2015 by the Laboratório de Flavivirus, IOC, Fiocruz. The isolate was grown in T25 flasks in Leibovitz medium (L-15) (Invitrogen) supplemented with 10% FBS (Gibco) and maintained at 28ºC. The sample was tested by RT-qPCR^15^ using specific primers, and the complete genome sequence was deposited in GenBank under accession number KX197205. The viral titre was 2.8 x 10^8^ PFU/mL, and the viral stocks were stored at -80ºC.

*Chikungunya virus* - The CHIKV sample used was isolated from human serum in Brazil (strain BHI-3737) in 2014. An aliquot of CHIKV was kindly supplied by Seção de Arbovirologia e Febres Hemorrágicas of the Evandro Chagas Instituto - IEC, Belém. It was reconstituted from a virus aliquot by the Laboratório de Mosquitos Transmissores de Hematozoários/LATHEMA, IOC/Fiocruz, and passaged in C6/36 cells grown in T25 flasks in L-15 medium supplemented (Invitrogen) with 10% FBS (Gibco) at 28ºC to produce large quantities of infected supernatant. The supernatant viral stock was stored at -80ºC, and the titre was 6.2 x 10^3^ PFU/mL.

Cell infections

*Aedes albopictus mosquito cell lineage (C6/36 cells)* - The virus samples (DENV-1, -2, -3, -4, ZIKV, CHIKV and YFV) were inoculated onto C6/36 cell monolayers cultivated in T25 flasks at an MOI of 1 and incubated for 1 h at 28ºC for virus adsorption. The monolayers were maintained in L-15 medium (Invitrogen) supplemented with 1% nonessential amino acids (Invitrogen), 5% FBS (Gibco), and 1% penicillin/streptomycin (P/S) (Invitrogen) at 28ºC. Cytopathic effects (CPEs) were investigated at 24, 48 and 72 h post infection (p.i.) by inverted light microscopy.

*Kidney epithelial cells of African green monkeys (Vero cells)* - ZIKV and CHIKV samples were inoculated onto Vero cell (CCL-81) monolayers cultivated in T25 flasks at an MOI of 1 and incubated for 1 h at 37ºC for virus adsorption. After the incubation period, the monolayers were maintained with Earle’s 199 (Invitrogen) medium supplemented with 5% FBS (Gibco), 2 mM glutamine and 100 U/50 μg P/S (Invitrogen) under an atmosphere containing 5% CO_2_ and incubated at 37ºC. CPE was investigated at 24, 48 and 72 h p.i. by inverted light microscopy.

Cell processing for transmission electron microscopy analysis

Cells were fixed in 1% glutaraldehyde in sodium cacodylate buffer (0.2 M, pH 7.2) [electron microscopy science (EMS)], post fixed in 1% buffered osmium tetroxide (EMS), dehydrated in acetone (Merck), embedded in epoxy resin (EMS) and polymerised at 60ºC for three days. Ultrathin sections (50-70 nm thick) were obtained from the resin blocks. The sections were placed onto copper grids, stained with uranyl acetate (EMS) and lead citrate (EMS), and observed in a Jeol JEM 1011 transmission electron microscope of the Plataforma de Microscopia Eletrônica, Fiocruz, Rio de Janeiro, Brazil.

## RESULTS

Flaviviruses: DENV, YFV and ZIKV

**Fig. 1:**
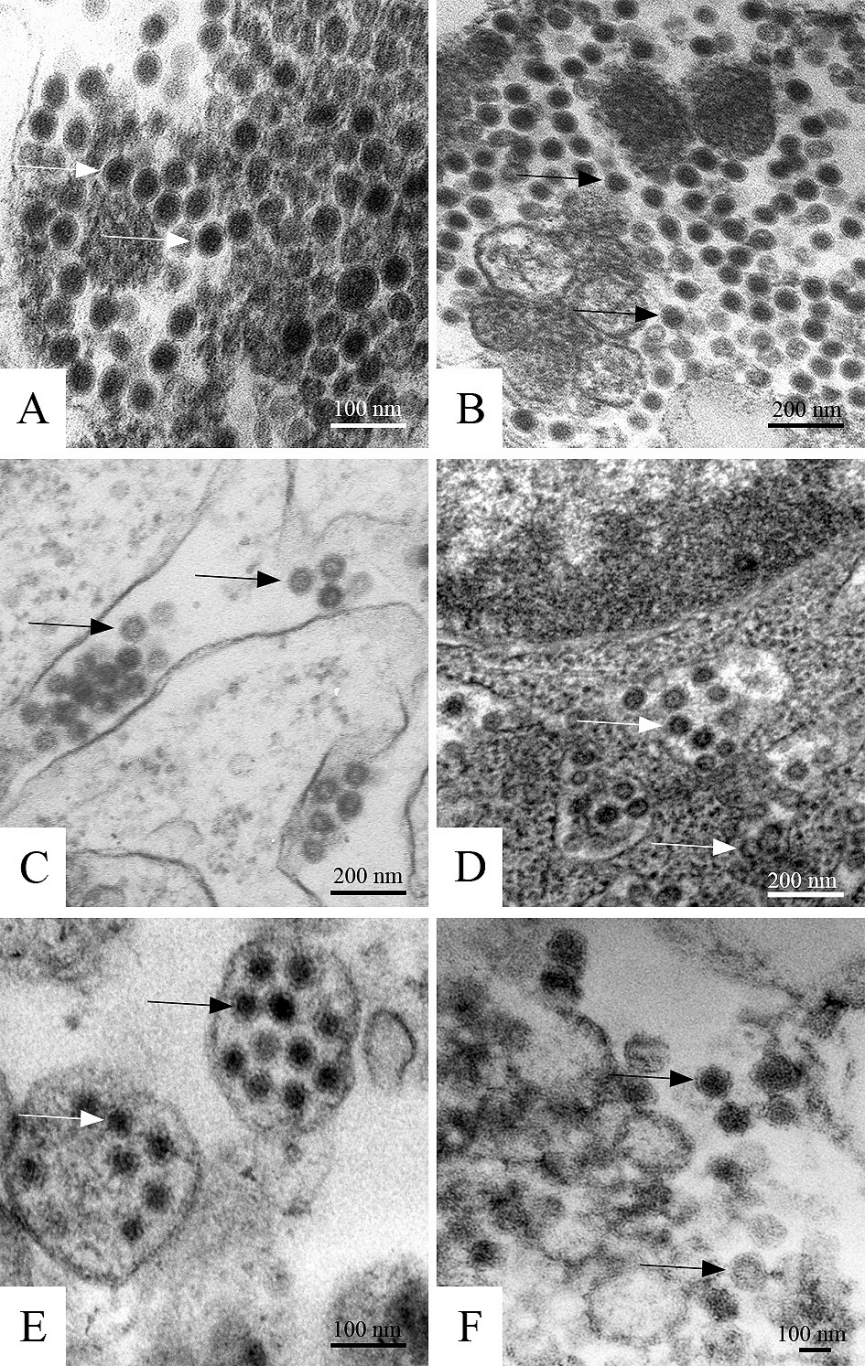
dengue, yellow fever and Zika virus particles (arrows) inside cytoplasmic vesicles of C6/36 and Vero cells analysed by transmission electron microscopy. (A) Dengue virus (DENV) serotype 1 (C6/36 cells, 72 h post infection), (B) DENV serotype 2 (C6/36 cells, 72 h post infection), (C) DENV serotype 3 (C6/36 cells, 72 h post infection), (D) DENV serotype 4 (C6/36 cells, 72 h post infection). The four serotypes of dengue particles were approximately 60 nm in diameter. (E) Yellow fever virus particles with an approximate diameter of 70 nm (C6/36 cells, 7 days post infection), (F) Zika virus particles approximately 40-50 nm in diameter (Vero cells, 48 h post infection).

*Virus particles morphology (* *Fig. 1* *)* - Spherical characteristic flavivirus particles consistent with virus replication were observed in cell cultures at all times of infection. The enveloped DENV particles showed an approximate diameter of 60 nm (Fig. 1A-D). Cells infected with YFV showed enveloped viral particles with an approximate diameter of 70 nm (Fig. 1E), and ZIKV particles were approximately 40-50 nm in diameter (Fig. 1F). The particle morphology of the four serotypes of DENV, YFV and ZIKV was similar.

**Fig. 2:**
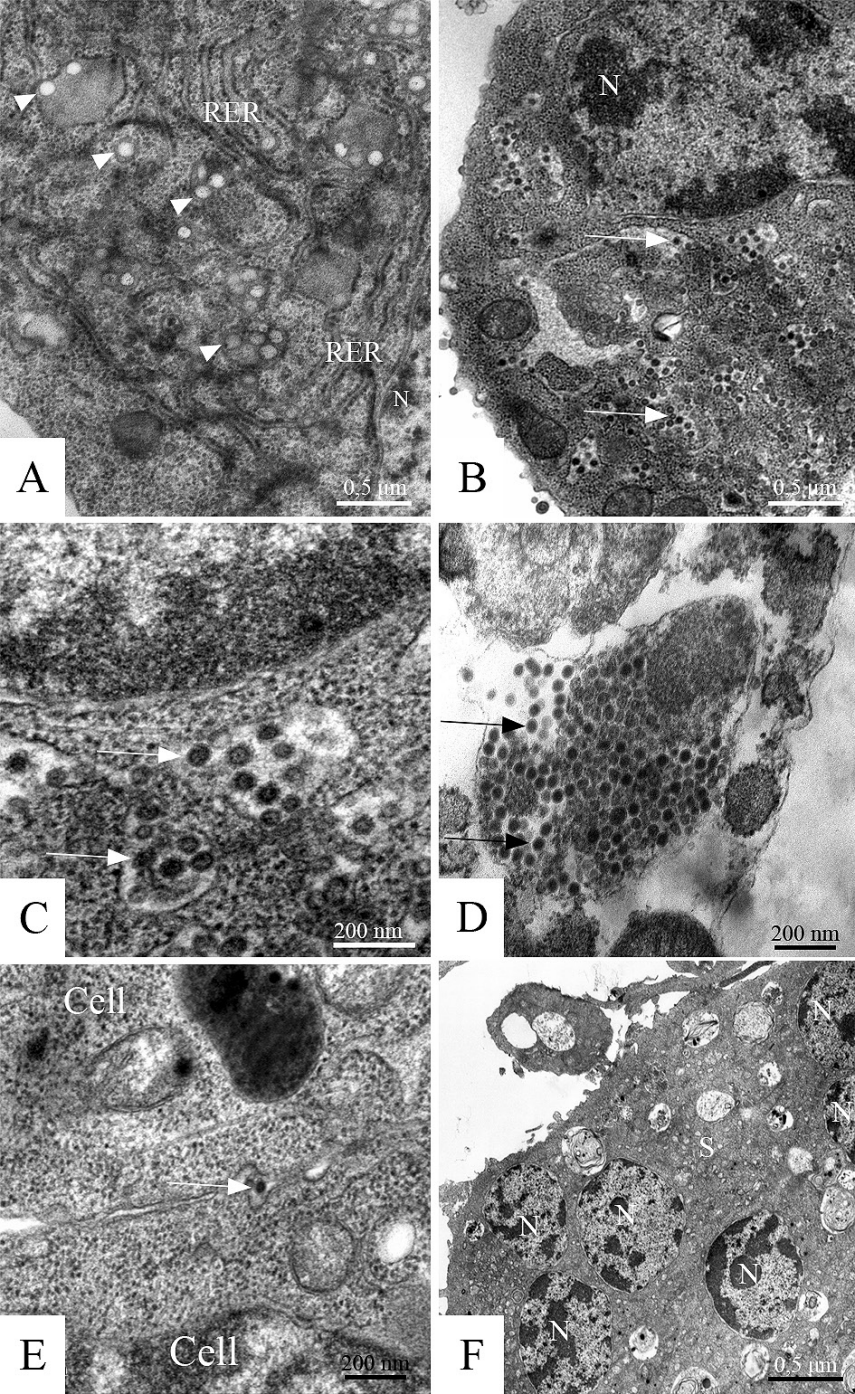
dengue virus (DENV). C6/36 cells 72 h post infection, transmission electron microscopy. (A) Cell infected with DENV serotype 1 presenting tubular structures (arrowhead) associated with the rough endoplasmic reticulum (RER), (B/C) DENV serotype 4 particles inside cytoplasmic vesicles (arrows), (D) DENV serotype 1 inside cytoplasmic vesicles (arrows), (E) DENV serotype 1 (arrow) intermediate the fusion of two cells, (F) syncytium (S). Nucleus (N).

**Fig. 3:**
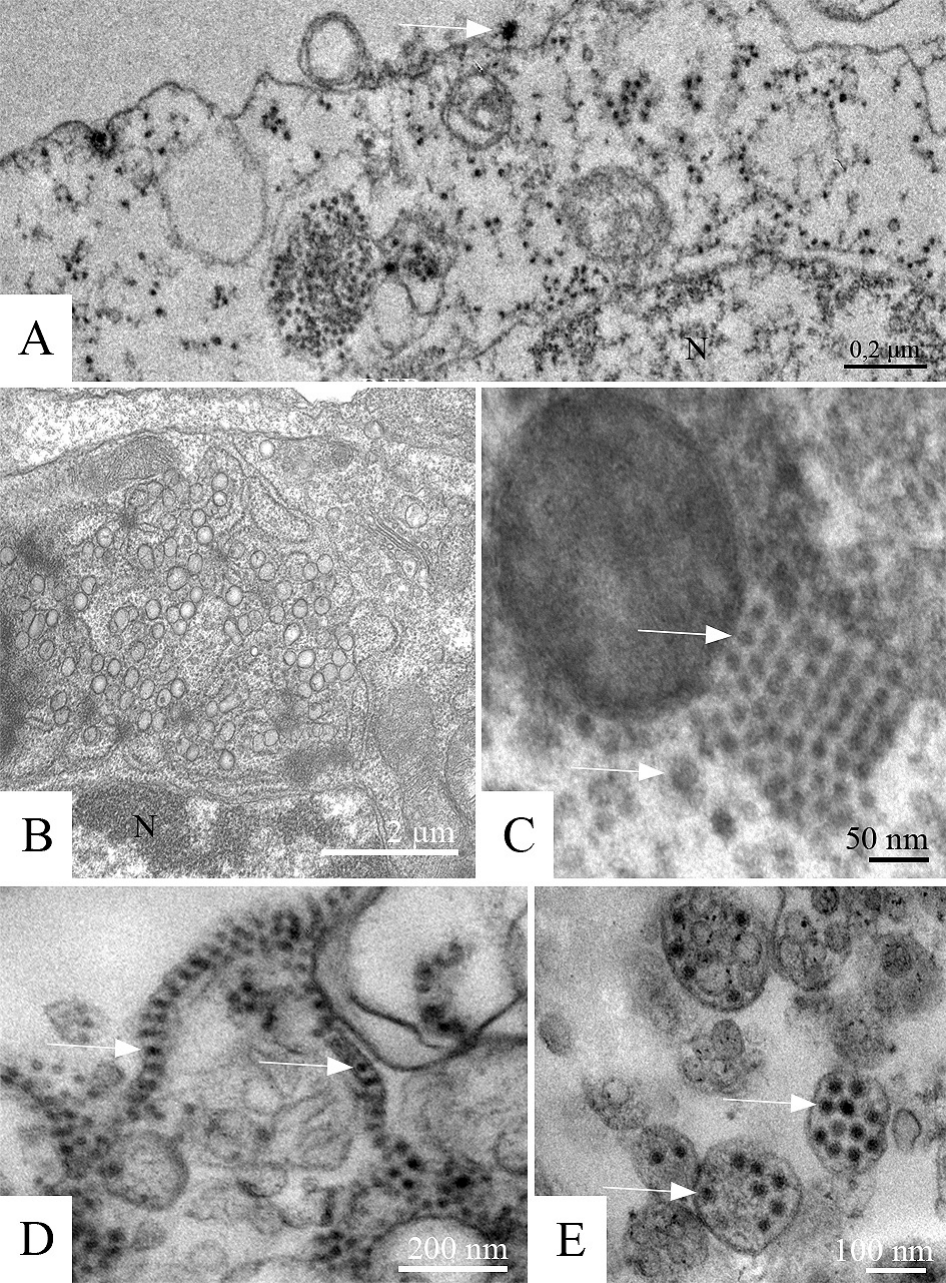
yellow fever virus (YFV). C6/36 cells seven days post infection, transmission electron microscopy. (A) One YFV particle interacting with cellular receptors (arrow) and another one being internalised through an endocytic vesicle coated with clathrin (arrowhead), (B) cell presenting tubular structures (asterisk) associated with the rough endoplasmic reticulum with YFV particles-like inside (arrow) with a diameter of approximately 200 nm, (C) YF nucleocapsids, (D) YFV particles inside cisterns of the rough endoplasmic reticulum (arrow), (E) YFV inside cytoplasmic vesicles (arrow). Nucleus (N).

**Fig. 4:**
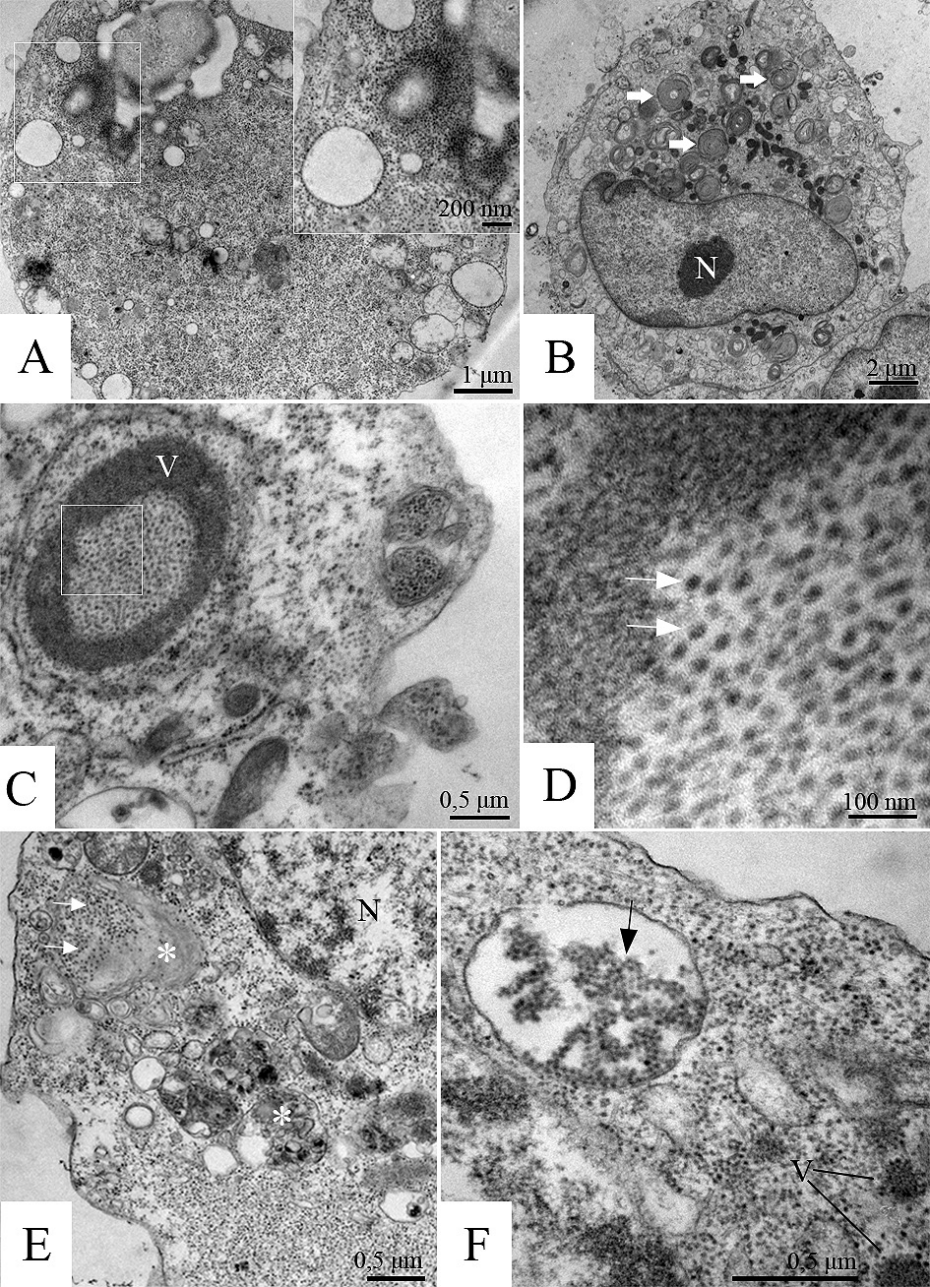
Zika virus (ZIKV). Infected C6/36 and Vero cells, transmission electron microscopy. (A) Thickened ribosomes (marked area) (C6/36 cells, 24 h post infection), (B) numerous myelin figures and phagosomes (arrow) (Vero cells, 72 h post infection), (C) viroplasm-like perinuclear compartment (V) containing ZIKV particles in their lumen (arrow) (C6/36 cells, 48 h post infection), (D) inset of marked area of the Figure (C, E) ZIKV particles (arrow) in lysosomes (asterisk) (C6/36 cells, 48 h post infection), (F) ZIKV particles inside an rough endoplasmic reticulum (RER) vesicle (arrow) (C6/36 cells, 48 h post infection). Nucleus (N), mitochondria (M).

*Virus morphogenesis (Figs 2-4)* - The replication of DENV-1, -2, -3 and -4, YFV and ZIKV in the cell monolayer was detected at all times of infection.

The internalisation of the viral particles in C6/36 cells infected with DENV-1, -2, -3 and -4 and YFV was observed early inside endocytic vesicles coated with clathrin (Fig. 3A). Later, the infected cells presented spherical and tubular structures (Figs 2A, 3B), and in the sequence, it was possible to observe viral particles during the assembly process (nucleocapsids, Fig. 3C) and particles already presenting an envelope inside cisterns of the rough endoplasmic reticulum (Figs 2B-C, 3D), in cytoplasmic vesicles (Figs 2D, 3E) and between the membranes of two cells in a fusion process (Fig. 2E). The cytopathic effect showed syncytia (Fig. 2F).

C6/36 and Vero cells infected with ZIKV showed an increased number of ribosomes (Fig. 4A), myelin figures, and numerous phagosomes (Fig. 4B) and lysosomes (Fig. 4D). Thickening of the nuclear membrane and vesicular compartments, measuring approximately 100 nm in diameter and associated with the rough endoplasmic reticulum were observed (data not shown). Large viroplasm-like compartments (Fig. 4C-D), localised in the perinuclear area, together with peripheral rough endoplasmic reticulum, mitochondria and microtubules, were verified. ZIKV particles were observed inside lysosomes (Fig. 4E).

Alphavirus: Chikungunya

**Fig. 5:**
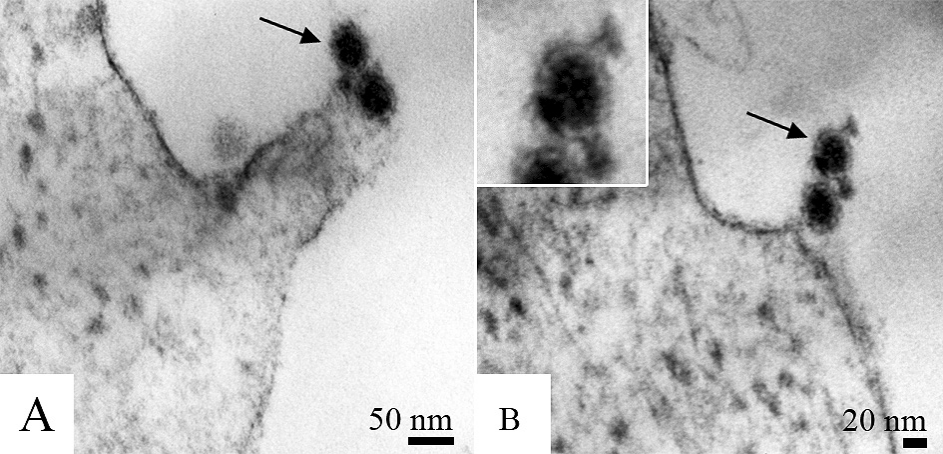
Chikungunya virus particles (arrow), Vero cells 72 h post infection, transmission electron microscopy. (A) Two viral particles budding from the cell membrane (arrow), (B) virus particles attached to the cell membrane (arrow); inset: higher magnification showing virus particles and their envelopes (arrowheads).

*Virus particles morphology (* *Fig. 5* *)* - Ultrastructural analysis showed virus particles and their replication in C6/36 and Vero cells when inoculated with CHIKV. The CHIKV particles have a spherical shape with an approximate diameter of 50-60 nm and an evident envelope structure (Fig. 5A-B).

**Fig. 6:**
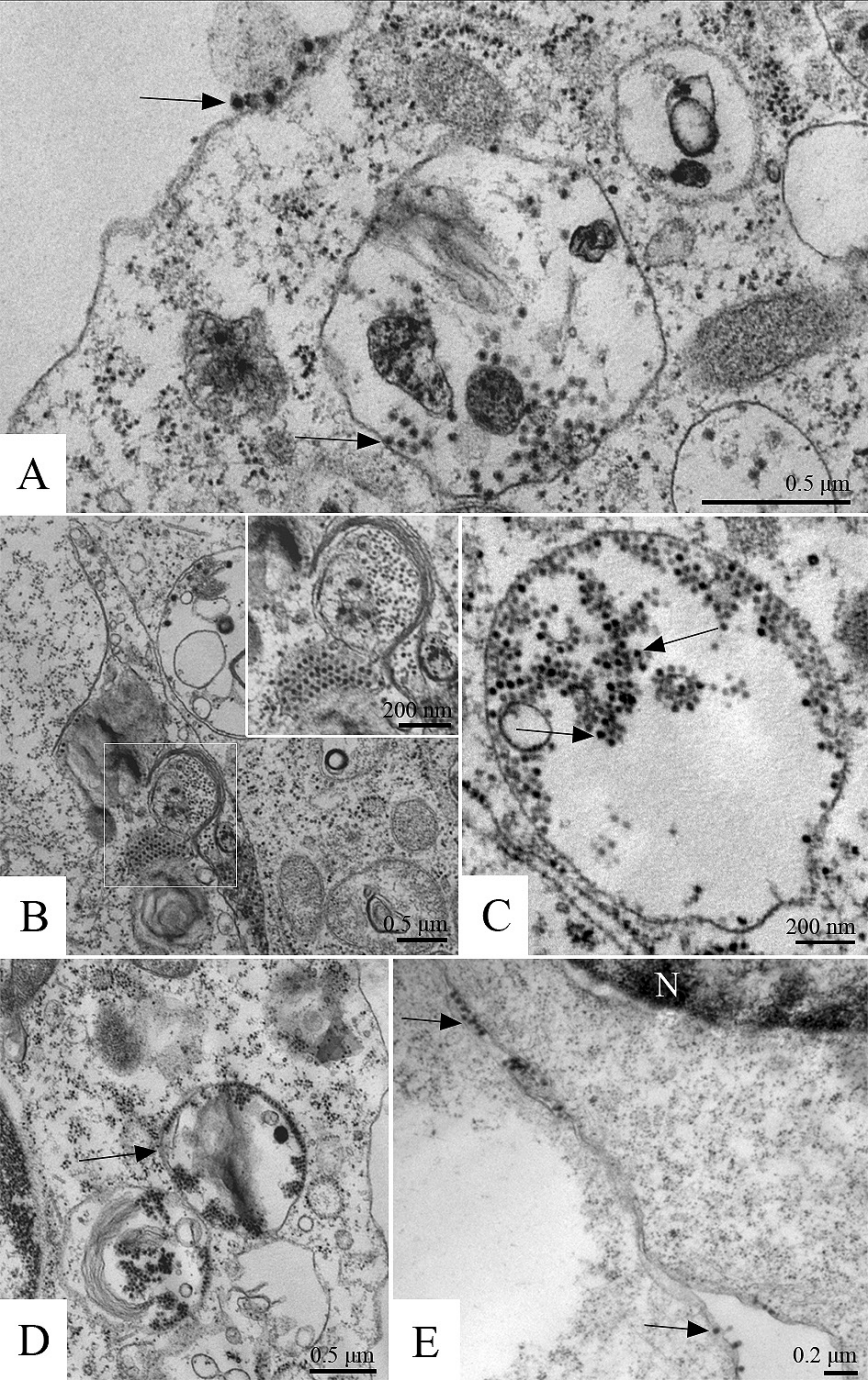
Chikungunya virus (CHIKV). C6/36 and Vero cells 72 h post infection, transmission electron microscopy. (A) Particles of CHIKV attached to the cell membrane (arrow) and inside a cell vesicle (asterisk) (C6/36 cell), (B) nucleocapsids in the cytoplasm (marked area), (C6/36 cells), (C/D) virus particles (arrows) associated with cell membranes (C6/36 cells), (E) viral particles budding from the cell membranes (arrows) (Vero cells). Nucleus (N).

*Virus morphogenesis (* *Fig. 6* *)* - C6/36 and Vero cells infected with CHIKV showed that the virus particles were internalised mainly by clathrin-mediated endocytosis (Fig. 6A). Viral nucleocapsids were detected inside and outside the vesicles (Fig. 6B). Virus particles occur inside unit-membrane coated cell compartments (Fig. 6C-D). The release of CHIKV particles by budding from the cell membrane was observed (Fig. 6E).

## DISCUSSION

The simultaneous circulation of different arboviruses (DENV, ZIKV, YFV and CHIKV) in Brazil is of extreme concern to public health since the effect of the cocirculation and coinfection of these viruses on the severity of the diseases they cause needs to be further investigated. Many arboviruses are endemic to Brazil, and recently, several coinfection cases have been reported. The morphological ultrastructural aspects and morphogenesis of these arboviruses currently circulating in Brazil were analysed using transmission electron microscopy.

The DENV infection cycle initiates virus attachment to the target cell through the interaction between viral surface proteins and attachment/receptor molecules on the cell surface. This interaction allows the internalisation of the virus particle, generally involving receptor-mediated endocytosis.^16^ ^,^ ^17^ The present study demonstrated that DENV, YFV and ZIKV attachment is similar to that of other flaviviruses (Figs 3A, 6A), and internalisation of the viral particle was observed early inside endocytic vesicles coated with clathrin (Fig. 3A). The DENV-1, -2, -3 -4, YFV and ZIKV particles presented a morphology and diameter similar to those observed in previous studies.^18^ ^,^ ^19^ ^,^ ^20^ ^,^ ^21^ No morphological differences were observed between the DENV serotypes.

In the present study, C6/36 and Vero cells infected with DENV-1, -2, -3 and -4 (Fig. 2A) and ZIKV presented spherical and tubular structures associated with rough endoplasmic reticulum and viral particle ontogenesis inside rough endoplasmic reticulum cisterns, as illustrated for DENV-2 in Barth 2000.^18^ This is in agreement with other reports about flaviviruses such as DENV, West Nile virus, Japanese encephalitis virus, tick-borne encephalitis virus and ZIKV.^8^ ^,^ ^22^ ^,^ ^23^ ^,^ ^24^ ^,^ ^25^ ^,^ ^26^ ^,^ ^27^ ^,^ ^28^ ^,^ ^29^ ^,^ ^30^ ^,^ ^31^ These tubular structures with an inner vesicle diameter of approximately 200 nm were observed in C6/36 cell cultures infected with YFV (Fig. 3E). Since DENV tubular structures were shown to be associated with dsRNA,^29^ ^,^ ^31^ these structures are believed to be the site of RNA replication. Single-stranded positive-sense RNA viruses are known to have properties that deform the endoplasmic reticulum membrane and create organelle-like compartments called viral replication organelles.^32^ ^,^ ^33^ Studies using electron microscopy found endoplasmic reticulum-derived DENV-induced vesicles, convoluted membranes, and virus particles and detected double-stranded RNA, a presumed marker of RNA replication, in addition to virus-induced vesicles and tubules. Electron tomography showed DENV-induced membrane structures to be part of an endoplasmic reticulum-derived network.^30^ Knowledge of cellular and viral factors participating in distinct steps of the flavivirus replication cycle is significant for the development of preventive and therapeutic strategies for combating dengue.

The flavivirus viral genome is a single-stranded positive-sense RNA that functions as a messenger RNA and is subsequently translated by the cell machinery, thus generating viral proteins. Afterward, the genome is replicated, and new RNA copies are incorporated into nascent particles. The assembly of DENV occurs inside the rough endoplasmic reticulum, and virions are then transported through the trans-Golgi network and secreted.^16^ ^,^ ^17^ These studies were corroborated in the present study, since DENV-1, -2, -3, -4 and YFV presented enveloped nucleocapsids inside the cisterns of the rough endoplasmic reticulum (Figs 2B-C, 3D) and in cytoplasmic vesicles (Figs 2D, 3E).

C6/36 and Vero cells infected with ZIKV showed large viroplasm-like compartments (Fig. 4C) localised in the perinuclear area next to the peripheral rough endoplasmic reticulum and microtubules. At the same time, large amounts of ribosomes and mitochondria were recruited. ZIKV particles inside lysosomes (Fig. 4D) and the rough endoplasmic reticulum (Fig. 4F), as well as viral nucleocapsids inside the viroplasm-like structures (Fig. 4C), were observed. These findings are similar to those observed by,^8^ demonstrating the susceptibility of C6/36 and Vero cells to ZIKV infection and that part of the replicative cycle might occur within viroplasm-like structures. This has not been previously observed in other flavivirus-infected cells.

Subsequent studies demonstrated that ZIKV infection in both human hepatoma and neuronal progenitor cells induced drastic structural modifications of the cellular architecture.^26^ ZIKV infection causes a drastic reorganisation of microtubules and intermediate filaments, forming cage-like structures surrounding the viral replication factories. Viroplasms are constituted during virus morphogenesis, when components such as replicase enzymes, virus genetic material, and host proteins are required for replication, thereby increasing the efficiency of replication.^34^ Viral replication, protein synthesis and assembly require a considerable amount of energy provided by large clusters of mitochondria at the periphery of viroplasms. The virus factory is often enclosed by a membrane derived from the rough endoplasmic reticulum or by cytoskeletal elements.^35^ Viroplasms have been observed in cauliflower mosaic virus, rotavirus, vaccinia virus, rice dwarf virus^36^ ^,^ ^37^ ^,^ ^38^ and mimivirus and reovirus.^39^ Further studies should be undertaken to understand the role of the viroplasm-like compartment in the ZIKV replication cycle.

C6/36 and Vero cell cultures inoculated with CHIKV internalised the virus particles mainly by clathrin-mediated endocytosis (Fig. 6A), as reported in other studies.^40^ ^,^ ^41^ ^,^ ^42^ Viral particle replication seems to occur inside unit membrane-coated cell compartments (Fig. 6C-D). Studies have demonstrated that negative-strand synthesis is linked to the formation of viral replication units termed spherules, which are small, vesicular structures that form at the plasma membrane and serve as the site of genomic viral RNA replication. The nonstructural proteins are thought to localise at the neck of the spherules, which house dsRNA intermediates, protecting them from degradation and recognition by cellular pattern-recognition receptors.^43^ ^,^ ^44^ ^,^ ^45^ ^,^ ^46^ ^,^ ^47^ ^,^ ^48^ As infection proceeds, the spherules are internalised to form large cytopathic vacuoles, which contain endosomal and lysosomal markers.^45^ ^,^ ^46^ ^,^ ^49^ The release of virus particles occurs by budding from the cell membrane,^50^ which was also observed in our studies (Fig. 6E).

Here, it was demonstrated that the morphology and morphogenesis of the currently circulating arboviruses (DENV, ZIKV, YFV and CHIKV) in Brazil are similar to what has been reported in previous studies. However, we understand that there need to be more studies to determine the role of the viroplasm-like structures in ZIKV replication.
